# Robotic System for Inspection by Contact of Bridge Beams Using UAVs †

**DOI:** 10.3390/s19020305

**Published:** 2019-01-14

**Authors:** Pedro J. Sanchez-Cuevas, Pablo Ramon-Soria, Begoña Arrue, Anibal Ollero, Guillermo Heredia

**Affiliations:** Robotics, Vision and Control Group, Universidad de Sevilla, Camino de los Descubrimientos s.n., 41092 Sevilla, Spain; prs@us.es (P.R.-S.); barrue@us.es (B.A.); aollero@us.es (A.O.); guiller@us.es (G.H.)

**Keywords:** robotic bridge inspection, UAS applications, aerial robotics, aerial inspection, aerodynamic ceiling effect

## Abstract

This paper presents a robotic system using Unmanned Aerial Vehicles (UAVs) for bridge-inspection tasks that require physical contact between the aerial platform and the bridge surfaces, such as beam-deflection analysis or measuring crack depth with an ultrasonic sensor. The proposed system takes advantage of the aerodynamic ceiling effect that arises when the multirotor gets close to the bridge surface. Moreover, this paper describes how a UAV can be used as a sensor that is able to fly and touch the bridge to take measurements during an inspection by contact. A practical application of the system involving the measurement of a bridge’s beam deflection using a laser tracking station is also presented. In order to validate our system, experiments on two different bridges involving the measurement of the deflection of their beams are shown.

## 1. Introduction

In recent years, interest in Unmanned Aerial Vehicles (UAVs) has grown exponentially [1]. These aerial robots have been used in a huge variety of applications, such as detection, exploration, monitoring, precise localization, or assistance in natural catastrophes. Nevertheless, in most of these applications, aerial robots have merely a perceptive role, carrying cameras and other optical sensors for environment sensing. Thus, these robots do not have any physical interaction with the environment.

Recently, the development of autonomous aerial robots capable of physically interacting with the environment is attracting the interest of researchers in the aerial robotics field [2,3]. For example, some researchers have embedded small and lightweight serial manipulators [4,5,6], offering a new innovative solution for applications such as the inspection and maintenance of industrial facilities or aerial power lines. The authors of Reference [7] proposed the use of UAVs for the assessment of post-disaster situations such as earthquakes.

The inspection of reinforced concrete bridges involves different tasks that are performed by inspectors to obtain information for the structural assessment of bridges. Inspectors usually employ ladders, scaffolding, or lifters to reach the parts of beams and piers of the bridge that are not easily accessible. One of the tasks is visual inspection in order to detect cracks, rebar corrosion, delamination, and other defects. Other tasks require direct contact of the sensor or device with the bridge surfaces. One of these contact tasks is the measurement of beam deflection with and without load, which gives information on the internal state of the bridge. This is usually done with an operator manually placing a reflector prism attached to a pole in contact with different points at the beam and then measuring the position of the prism with a laser total station [8]. Other tasks that require contact are the measurement of crack depth with an ultrasonic sensor [9], and the measurement of crack width with tactile sensors [10].

Previous studies proposed autonomous data-collection systems to overtake the less-efficient and costlier current method of manual bridge inspection, including novel methods to automatically assess cracks based on the combination of image processing and terrestrial laser scanning [11]. The authors of Reference [12] described the use of a robotized system for autonomously acquiring data from the top of a bridge to evaluate its status with nondestructive techniques. Some studies have proposed solutions using Unmanned Ground Vehicles (UGVs) for robotic crack inspection and mapping [13], delamination and concrete quality assessment [14], and even a complete mechatronic system for high-efficiency bridge inspection [15]. The authors of Reference [16] also presented a semi-autonomous robotic system focused on road and railway bridges.

The use of UAVs for bridge inspection is not as extended as the use of ground vehicles, but has increased significantly in previous years [17]. UAVs have been mostly used for the visual inspection of bridges [18], since standard UAVs have to fly at a safe distance from bridges.

**Figure 1:**
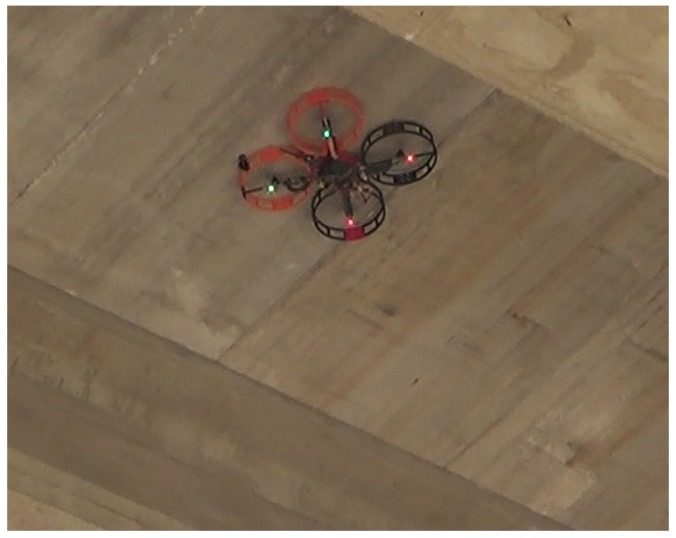
Prototype of multirotor system operating in contact with the ceiling.

Aerial manipulators are usually composed of an aerial platform and an articulated robotic arm. The authors of References [19,20] developed a system with an arm attached to the top of the multirotor, which was used to conduct inspection tasks that require contact with the bridge. However, payload requirements for this kind of manipulator are high, resulting in large and heavy platforms that are slower and more complex to control. This paper proposes a small and lightweight multirotor design that is capable of flying close to the bridge and sticking to it thanks to the aerodynamic ceiling effect to perform contact inspection tasks (Figure 1). This aerial robot was designed under the framework of the AEROBI European Project [21], which aims to develop an aerial robotic system that can perform both visual and contact bridge inspection tasks.

Airflow generated by rotary wings is sensitive to the physical obstacles close to it. This modification of the airflow changes the resulting thrust and torque generated by the robot’s engines. This effect can be observed when UAVs take off, land, or even fly at very low altitude [22,23]. The presence of the ground perturbs the natural motion of the air, known as the ground effect. This effect is characterized by the production of an increment in the thrust generated by the rotors without increasing the power. A similar effect is caused by flat surfaces placed above the rotors, known as the ceiling effect. This phenomenon induces an additional thrust on the robot, pushing it towards the ceiling. This fact can be dangerous for standard multirotors because propellers and engines may collide with the ceiling and break, or destabilize the platform.

This paper describes an integrated solution using a laser-tracking station and a UAV that are able to carry out inspection tasks. Thanks to the ceiling effect, the UAV is able to remain in contact with the bridge deck, and beams from below using a protective frame. This frame acts as armor that protects rotors when the robot is in contact with the beams, taking advantage of the ceiling effect. Meanwhile, a laser-tracking station in the ground accurately measures the position of a reflector prism mounted on the UAV. These measurements are later used to estimate bridge deflection.

The total station is an optical and electronic instrument used in building construction and surveying. This device can measure distances and horizontal/vertical angles from it to a single far away point. This point can be any surface or a reflector prism. Total stations are used by civil engineers to record features in topographic surveying. Archaeologists also use them to record excavations. Another situation in which it is essential to have environment reconstruction is in a crime scene or a traffic accident, where the police or insurance companies need to take measurements of scenes.

A short version of this paper with preliminary results was presented at ICUAS 2017 [24]. Extensive testing is presented in this paper, including the laser-prism tracking-system results, the effects on UAV magnetometer sensors while flying close to reinforced concrete structures, and experimental contact tests on different bridges.

The remainder of the article is organized as follows: Section 2 presents the general architecture of the proposed robotic bridge-inspection system. This shows the concepts of the system and the performance during the inspection. Section 3 presents the requirements of the aerial platform and the solution proposed for carrying out the bridge-beam inspection system. Section 4 focuses on the inspection sensors that are used in taking measurements of the bridge beam deflections. Section 5 is about the bridge-inspection experiments and the validation of the system. Lastly, conclusions and future works for this research are discussed.

## 2. Robotic Bridge-Inspection System

**Figure 2:**
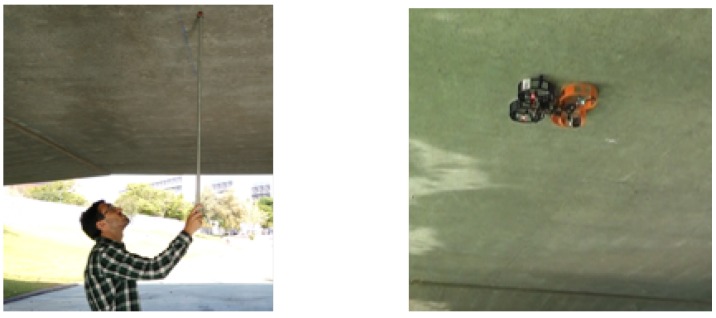
Inspection by manual contact task vs. with an Unmanned Aerial Vehicle (UAV).

This section presents robotic bridge inspection by a contact system using a UAV that is proposed in this paper. The system focuses on measuring the deflection of bridge beams using a UAV as a flying sensor that is able to touch the bridge in order to get the best possible measure with an aerial platform. Traditionally, deflection measurements of bridges have been carried out by a human operator who manually places the reflector prism at several points under the beam. Thus, this research proposes an autonomous system that uses a UAV with an inspection sensor onboard to make this task easier, faster, and safer for people (see Figure 2).

**Figure 3:**
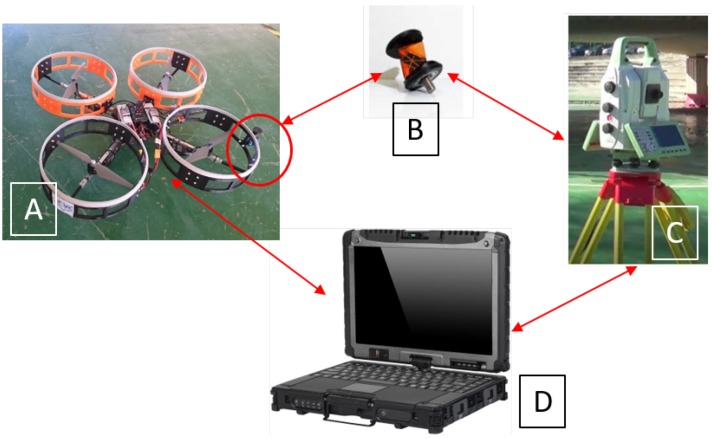
Robotics bridge-inspection system. (**A**) Aerial platform, (**B**) onboard reflector prism, (**C**) total station, and (**D**) ground-control system for monitoring and collecting flight and inspection data.

The inspection system is composed of an aerial platform, which has a reflector prism mounted onboard that acts as an inspection sensor, a ground-control station to monitor the state of the aircraft, and a laser-tracking station which follows the position of the reflector prism and takes the deflection measures of the bridge beams. Figure 3 shows the hardware architecture of this system. During inspection, the UAV is commanded to touch a specific point on the bottom surface of the bridge with the fairing of the rotors; this is the target inspection point. Once the multirotor establishes the contact with the bridge, it remains stuck to it during the inspection phase. Thus, the onboard reflector prism is also in contact with the bridge while the inspection task is carried out. Meanwhile, the total station takes several high-precision measurements using the position of the reflector prism mounted in the UAV. These measurements can be used to estimate the deflection of the bridge and the changes with respect to the bridge design. This concept presents the aerial platform as a flying sensor that can obtain high-precision measurements by putting the reflector prism in a target inspection point to perform the bridge inspection by contact. Moreover, one of the advantages of including a robotic total station and a reflector prism in this robotic inspection system is that it allows the establishment of a fixed reference frame over time so that the results obtained at different inspection points for each bridge can be compared. This is explained in detail in Section 5.

In summary, this robotic system is composed of two main parts, the aerial platform (on the left) and the total station (on the right), as well as the reflector prism. Each one is explained in its corresponding section below.

## 3. Aerial Platform

Bridge inspection usually requires high precision in taking measurements during the inspection task. This requirement imposes strong constraints from an aerial-platform design point of view. In this paper, an aerial robot that is able to touch and maintain contact with the beams during inspections under safe conditions, and prevents all movement, is proposed. Thus, there are no errors derived from the motion of the aircraft during the inspection. Moreover, the maximum size of the aerial platform is limited by the size of the bridge beams that are going to be inspected. The multirotor should fit in the beam width in a full contact condition, in which the entire upper body is touching the bridge. This section is about the design of the aerial platform and how this design can deal with the size and the contact constraints.

### 3.1. Ceiling Effect

As was mentioned in the previous section, the kind of inspection proposed in this paper requires that the multirotor flies very close to the bridge beams where the aerodynamic disturbance produced by the presence of a ceiling-type surface begins to be significant. This aerodynamic disturbance is known as the ceiling effect in the literature [25,26,27]. This ceiling effect has been studied by the authors and was modeled for a single-rotor working close to a ceiling surface in Reference [24] as follows:

(1) $$K_{c}\left(z\right)=\frac{T_{ICE}\left(z\right)}{T_{OCE}}=\frac{1}{1-\frac{1}{K_{1}}{\left(\frac{R}{z+K_{2}}\right)}^{2}}$$

where $T_{ICE}\left(z\right)/T_{OCE}$ is the ceiling factor $K_{c}\left(z\right)$ that models the changes between the thrust of a rotor, known as the “in-ceiling effect” ($T_{ICE}$), and the thrust of a rotor, known as the “out-ceiling effect” ($T_{OCE}$). *R* is the radius of the rotor, and *z* is the distance from the rotor to the ceiling. $K_{1}$ and $K_{2}$ are experimental coefficients obtained by the least-squares method as $K_{1}=6.924$ and $K_{2}=0.03782\left(m\right)$ for this aerial robot. This ceiling factor was presented by the authors in Reference [18].

### 3.2. Multirotor Design

In applications that need the UAV to fly very close to a surface with a sensor touching it, as is the case for concrete-bridge inspection with ultrasonic and laser sensors, it is challenging to maintain the stability of the UAV with sufficient positioning accuracy for taking measurements. Thus, this paper proposes the design of a custom multirotor that takes advantage of the ceiling effect to stick firmly to the bridge’s surface. In this way, the stability of the multirotor when taking measurements with the sensor is significantly improved. Another compelling advantage is that once the multirotor is stuck to the bridge, the rotation speed of the rotors can be lowered, reducing energy consumption and increasing flight time due to the ceiling effect. However, most aerial platforms are not designed to maintain contact with a surface with their upper body. Some of them may touch the surface with the propellers, leading to dangerous flight conditions, and others have antennas or sensors that stand out without any protection. Therefore, it is necessary to design a new multirotor configuration that can safely touch the bridge surface, maintain contact during the inspection, and provide protection from impacts to the upper part and the propellers of the aircraft.

**Figure 4:**
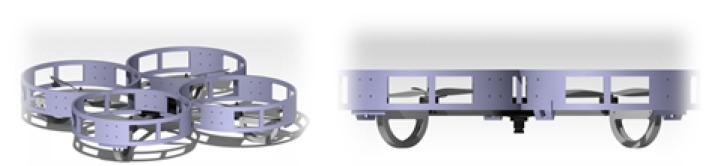
3D Design of the platform.

The multirotor proposed in this paper is based on a cross-layout quadrotor with a special fairing surrounding the propellers. All electronic components (autopilot, batteries, sensors, and electronics) are placed at the rotor’s plane or below it, as shown in Figure 4. This design allows the multirotor to safely maintain contact with the lower part of the bridge. The fairings are designed so that while in contact with bridge beams, the rotors spin a few centimeters from the surface without colliding with it. The proposed design sets this value to 0.36R.

A DJI E305 motor kit from the Chinese technology company SZ DJI Technology Co., Ltd. was selected with a power supply consisting of a 4s LiPo battery. This set delivers a nominal thrust of approximately 400–450 g per axis. The total weight of the platform is about 1.5 kg with a maximum flight time of 14 min. The fixed distance between the rotors axis is 480 mm, and the total size of the platform is about 600 mm, which fits most of the beams and complies with the inspection requirements.

The fairings surround each rotor with a cylindrical shape to ensure the safety of the propellers and the multirotor itself. The diameter of each fairing is 280 mm, and height is 74 mm. The top of the fairing is covered with a rubber material to prevent slippage on the ceiling surface and to dampen the contact with the surface. As can be seen in Figure 4, these fairings present air intakes around the circumference to ensure thrust is generated by the rotors when the multirotor is stuck to the ceiling. Moreover, this fairing isolates the aerodynamic effect between different rotors and allows the aerodynamic effect of the rotor to be modeled, assuming that it is working without any disturbance from the other rotors. This is because, as was presented by the authors in Reference [24], it was expected that there would not be much difference between the single-rotor and the complete-quadrotor cases. This was taken as a preliminary design to validate the presented prototype in this paper. Nevertheless, the optimization of the fairing design in order to improve the weight and the aerodynamic performance will be studied in future works. It is important to remark that the fairing makes the aircraft more sensitive to wind and limits the operation of the aircraft to wind speeds below 20 km/h.

**Figure 5:**
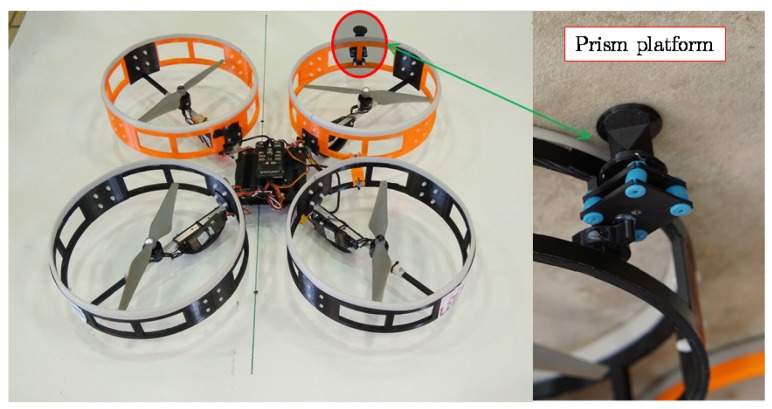
Final prototype of the platform and prism platform detail.

Figure 5 shows the final prototype of the quadrotor with the fairings surrounding the rotors. The fairings were built in polylactide (PLA) with 3D-printing technology. Front rotors were printed in orange and rear rotors in black.

The platform cannot carry out the bridge inspection by itself. So, this prototype has a custom socket to mount the inspection sensor onboard that is used by the laser system to accurately measure the position co-ordinates of each point in the bridge. The prism platform has four silent blocks that are compressed in the contact condition. These have two functions. First, they guarantee the contact of the prism because they overhang the fairings that, during contact, could be a bit compressed. Second, they adapt the prism contact to the bridge surface in cases where the bridge has a soft slope or small defects.

### 3.3. Other Effects in Sensors

In addition to the aerodynamic effects, other problems arise when an aircraft flies in narrow places or under structures like bridges. These problems are mainly related to the specific autopilot system that controls the aircraft and the constraints suffered by the integrated sensors onboard for position and attitude estimation. The main problems arise in the Global Positioning System (GPS) signal and the magnetometer measurements. GPS signals are partially or fully blocked under bridges. Hence, these signals cannot be used to feed the position estimator. Additionally, reinforced concrete-bridge armatures significantly change the magnetic field measured by the onboard magnetometers. Consequently, this measurement of the magnetometer cannot be used reliably for attitude estimation. This effect is better explained through telemetry data during flights in Section 4.

The experiments of this paper were carried out using the PX4 that is an open source flight stack obtained from [28]. However, some modifications had to be included in the estimation and the control algorithm. The main changes in the estimation module were due to mentioned constraints in the GPS and magnetometer and are extended below. Relative to the control part, it is necessary to remark that the position controller cannot use the GPS signal because it might not be available under the bridge. Instead, optical flow [29] or visual odometry [30] can be used for relative position estimation. Besides, during the contact, it is necessary to control the integral errors of the attitude controller because the multirotor is never in contact with a perfectly null tilt. Thus, if the integral attitude controller is on during contact, the separation maneuver can be very aggressive and dangerous due to the high number of integral errors.

The attitude estimator must be adjusted because of the errors in the magnetometer. This disturbance is caused by the possible metallic structure of the bridge or the presence of cables close to or inside it, among other reasons. Thus, the measurements of this sensor are very distorted while flying close to the bridge surface and this is tough to model, so it cannot be used to estimate the attitude of the multirotor. This fact mainly affects the accuracy of the yaw orientation angle estimation. Concerning the attitude control of the autopilot, only the yaw controller was modified to prevent the controller saturating due to error accumulation when the multirotor is stuck to the ceiling. This error accumulation arises in this condition because the multirotor cannot correct this error and starts to saturate the mixer. To guarantee that contact occurs under safe conditions, a yaw rate controller without an integral term is used during the contact.

## 4. Total Station and Reflector Prism

A total station is a robotized sensor used to measure distances with laser technology. It can measure the distance to any surface or a reflector prism using a highly accurate laser system. This tool is often used in topography and building construction [31]. The tool has a laser emitter and sensor that measures the distance to a point that can be oriented in azimuth and elevation angles. Using these angles and the laser measure, the total station triangulates the positions of the desired points in local axis, which are defined by the user, with high accuracy.

Even if the total station is able to measure the distance to any object or surface, when it works with the reflector prism, it generates more accurate measurements. There are several different types of prism, such as flat prisms, which are the most frequently used in topography (thought to work at larger distances), and the 360-degree reflector prisms. The latter are of high interest for UAV applications. The main advantage of this kind of prism is that it is not necessary to orient it in a specific direction, i.e., it reflects the laser beam in any direction in order to measure its position, although the accuracy is a bit lower.

**Figure 6:**
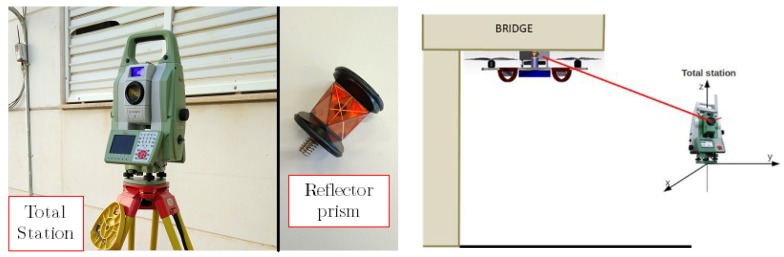
Total station and 360° mini-prism. (**Left**): actual laser system and the reflector mini-prism 360. (**Right**): scheme of the utilization of the laser tracker to obtain the UAV position.

The model used for this project was the MS50 from the Swiss company Leica Geosystem [31] (Figure 6). It has a theoretical accuracy of 1 mm for the prism and 2 mm for any surface. The maximum distance measurement is 3000 m for 360° prisms and 10,000 m for flat prisms. The prism used in this project was a 360° miniprism from Leica (Figure 6), which does not need to be oriented to obtain its position. To be more precise, the prism works at 360° in the vertical axis of the prism and, starting from the horizontal plane, it covers 60°.

The total station is placed on a tripod that allows it to level and produce reliable measurements. Then, the laser system can track the prism and continuously monitor it. This is possible thanks to a pair of high-accuracy motors with encoders that orientate the device towards the prism. The UAV trajectory can be obtained through the prism trajectory by a constant affine transformation that is designed with the overall design of the platform.

**Figure 7:**
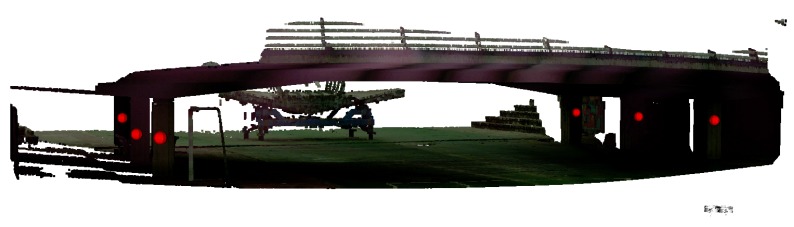
Scan of bridge and landmarks to determine consistency in the experiments.

Additionally, as the laser emitter can be used to measure the distance to any surface, the Leica Total Station can be used to acquire 3D laser scans of the bridges. However, these scans usually take long periods of time, as each point is captured individually. Additionally, the origin of the point cloud is located in the original position of the TS. This fact can be a problem for the inspection, because measurements need to be consistent over time. For this reason, each of the maps is always recorded with a set of landmarks that are used to reallocate the total station before each experiment. This means that it is not necessary to place the tool in the same place, but instead, each time the tool is placed, the relative transformation between the original location and the current one is computed by manually identifying the landmarks defined within the map. Thus, the 3D dense map can be reused, saving time in all the future experiments, and all the locations and measurements are consistent over time. Figure 7 shows an example of the 3D point cloud captured by the total station. Additionally, landmarks have been highlighted with large red spheres. The actual landmarks are single points in space.

**Figure 8:**
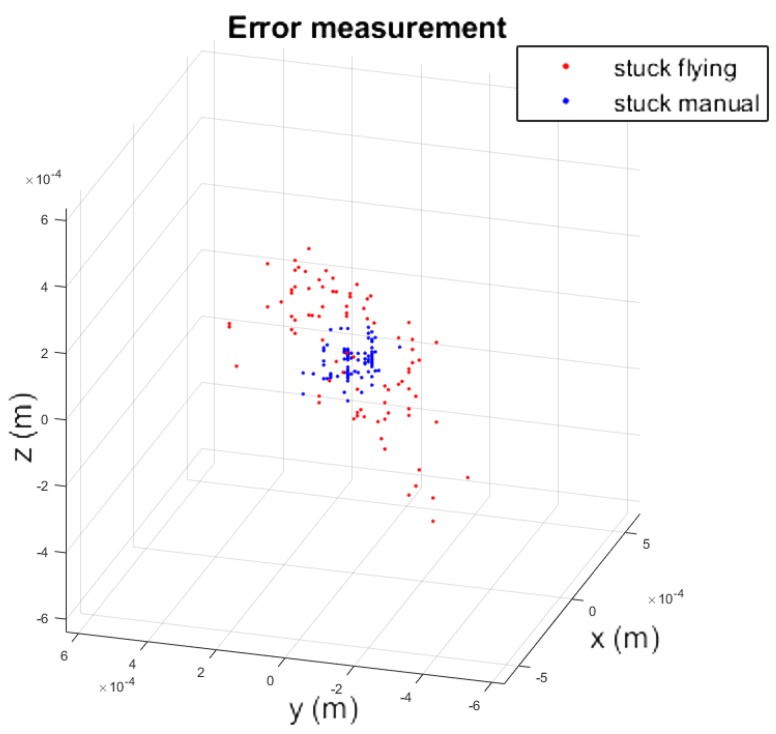
Comparison of the prism of the total station during flying inspection and manual inspection.

In order to give a quantitative analysis of the performance of the total station when the prism is built in the UAV during a flight, a set of measurements was carried out to compare the manual and aerial inspection, as shown in Figure 2. This experiment resulted in two scattered sets of points. To compare them, the mean of each of these sets was subtracted and these were plotted aside each other, as shown in Figure 8. It can be seen that the static measurement has less dispersion. Nevertheless, the variance of the experiment stuck while flying was 0.1 mm, with the maximum distance between any pair of points being lower than 1 mm.

These values are valid regardless of wind conditions, as long as they are moderate and allow the flight to be carried out under safe conditions, because during the contact phase, the platform can develop enough force to stay in contact with the ceiling and to prevent movement of the aircraft.

## 5. Experimental Validation

This section presents an experimental validation of this robotic system for bridge inspection by contact using UAVs. The experiments presented in this paper were carried out on two bridges with different features. These were the Cartuja and the Grazalema bridges.

**Figure 9:**
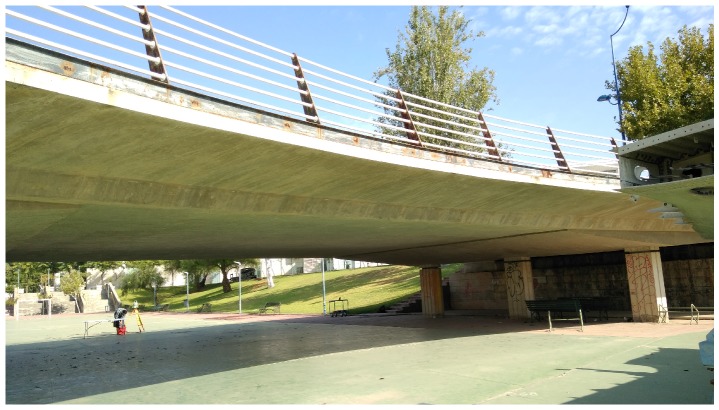
Cartuja bridge.

The Cartuja bridge (see Figure 9) has a reinforced concrete voided slab as its superstructure, which is totally flat on the bottom surface. The height of this bridge is approximately 4 m, and the substructure is made up of reinforced concrete piers with a rectangular section. This kind of bridge is the simplest type for carrying out inspection by contact due to its flat surface.

**Figure 10:**
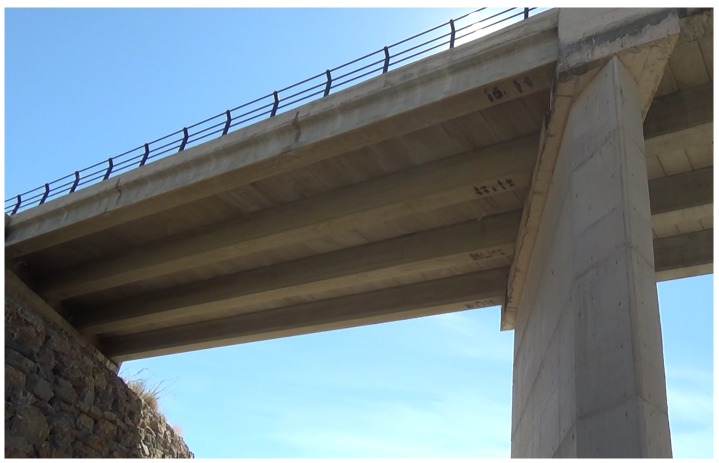
Grazalema bridge.

The Grazalema bridge (see Figure 10) has a reinforced concrete superstructure that is simply supported by I-beams. Because of this, the bottom surface of the bridge shows beams and spans (between the beams) at different height levels, and the inspection task is more complicated. The height of the bridge is around 14 m, and the substructure is made up of reinforced concrete walls.

**Figure 11:**
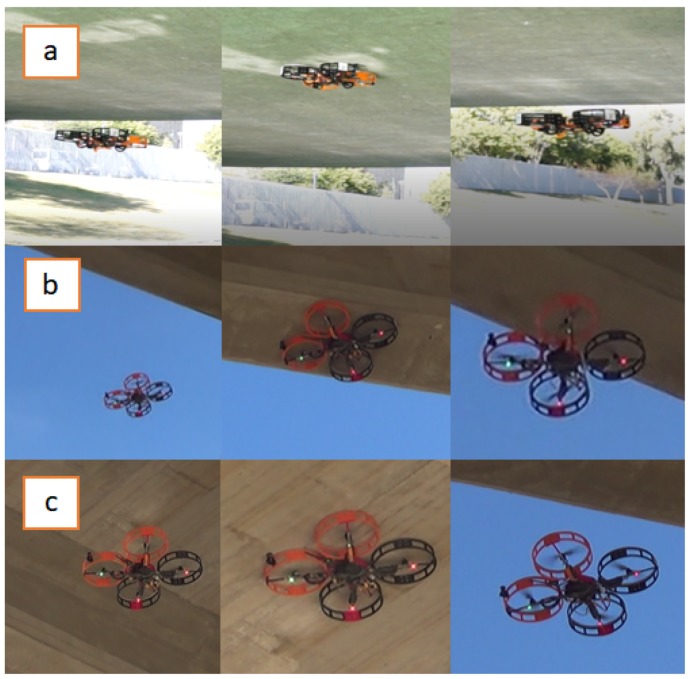
Sequences of the inspection by contact applications in three different kind of bridge. (**a**) Application for the Cartuja bridge (flat surface), (**b**) application for the Grazalema bridge (beam inspection), (**c**) application for the Grazalema bridge (gap inspection).

Figure 11 shows three sample sequences of the experiments with the proposed system for bridge inspection. The first sequence was carried out in the Cartuja bridge that has an entirely flat bottom surface (Figure 9). In the second and third sequences, the multirotor operated in the Grazalema bridge (Figure 10). In the second sequence, the multirotor inspected a bridge beam, and the third sequence shows that the aerial platform could also inspect the span between the beams.

These experiments validate the design and confirm the benefits of using the ceiling effect in the applications discussed in Section 2. The results of the experiments are shown in Section 5.1 and Section 5.2. A video compilation of the experiments can be found in Reference [32].

### 5.1. Cartuja Bridge Results

**Figure 12:**
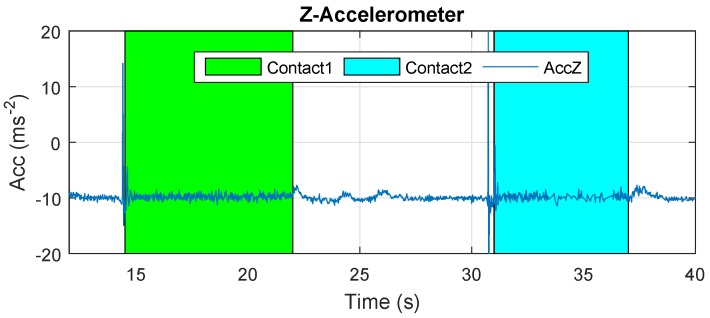
Z-accelerometer—Cartuja bridge experiments.

**Figure 13:**
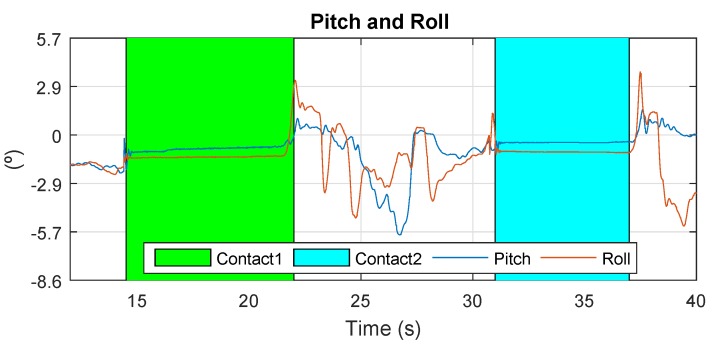
Pitch and roll—Cartuja bridge experiments.

**Figure 14:**
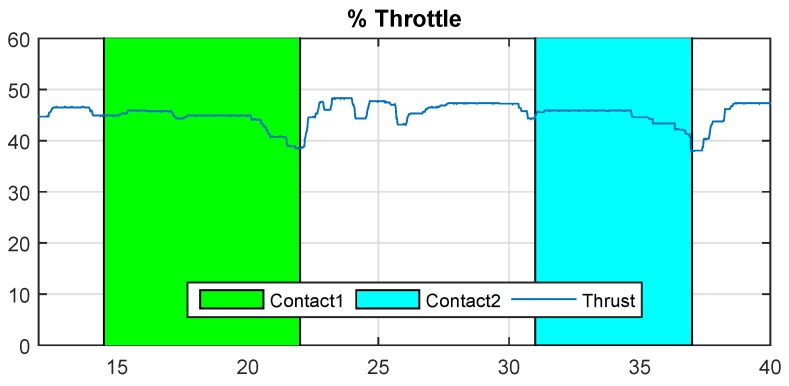
% Throttle—Cartuja bridge experiments.

**Figure 15:**
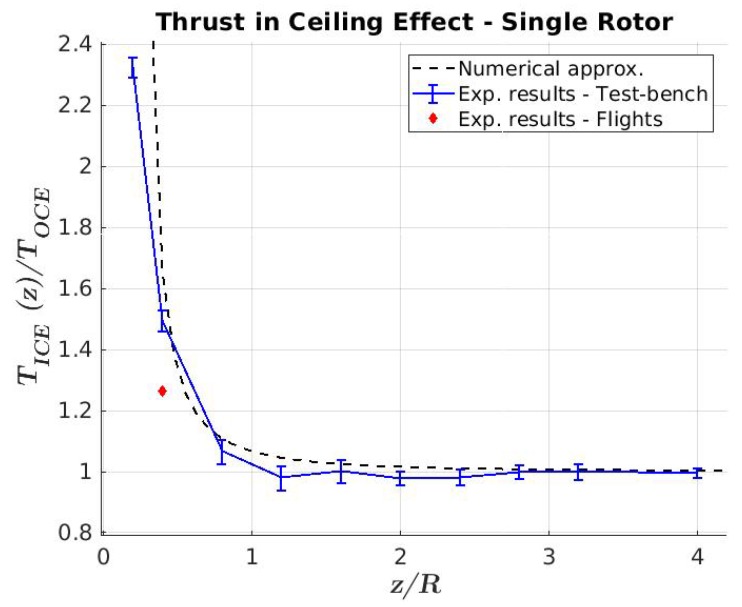
Ceiling-factor results. The blue error bar shows the experimental results obtained in a test bench, the black curve is the numerical approximation, and the red marker represents the ceiling factor obtained with the flight data.

**Figure 16:**
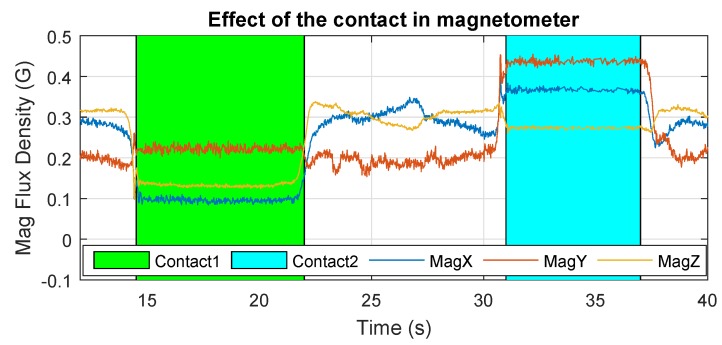
Magnetic-flux density—Cartuja bridge experiments.

**Figure 17:**
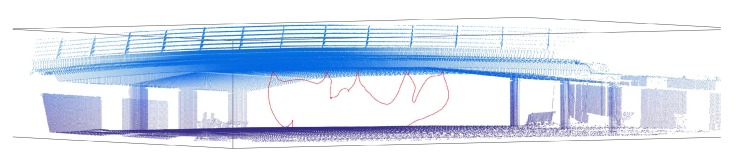
Prism trajectory measured with the laser tracker—Cartuja bridge experiments.

The results of Cartuja’s bridge-inspection task are presented in Figure 12, Figure 13, Figure 14, Figure 15, Figure 16 and Figure 17. These figures show an experiment in which the UAV first approaches the bottom surface of the bridge superstructure and then sticks to it. Later, it releases from the bridge and flies to a second point, coming in contact with the bridge and maintaining contact for several seconds. Finally, the UAV flies away from the bridge and lands. The first period of time in which the UAV is in contact with the bridge is colored in green in the figures, and the second contact period is colored in cyan. In both cases, the results are similar because the contact surface is flat and horizontal (see Reference [32]).

The presented results are a combination of the flight data and the laser-tracker data collected through telemetry and the laser tracker, respectively. The abscissa axes of these figures represent flight time in seconds.

The results presented in Figure 12 show that the z-accelerometer is triggered at the start of the contact condition due to the initial impact when contacting the bridge. Thus, this sensor can be used to detect the beginning of the contact condition. The design presented in this work guarantees that this impact is not a risk for UAV safety.

The next results are related to multirotor’s attitude during contact. In Figure 13, it can be observed that the angles remain steady while the multirotor is stuck to the bridge. During contact, the multirotor stays still, allowing the inspection task to occur without requiring attention to the control of the aerial robot. This is one of the most important results, because the fact that it stays in contact decreases the motion blur that UAVs can suffer during this type of operation.

Figure 14 shows the ceiling effect interference during contact. These experiments show that the thrust necessary to remain in contact is approximately 38%, which is significantly lower than the thrust needed in the hover condition (approximately 48%). This result is similar for the two contacts because the aerodynamic problem is similar in both cases.

The relation between the hover-thrust “in-ceiling effect” and “out-ceiling effect” during contact with the bridge is presented in Figure 15, which clearly shows that the aerodynamic ceiling effect is well-modeled with the expression (1).

Figure 16 shows the measurements of the magnetometer during the flight. It can be observed that the magnetic-flux density is greatly influenced by the metallic structure of the bridge, making the magnetic sensor unusable for attitude estimation. This provides evidence that the changes introduced in the autopilot (Section 3.3) are necessary to safely carry out these experiments.

The last figure presents the results of a complete inspection experiment. Figure 17 shows the trajectory of the prism measured by the laser tracker during the flight during the inspection task overlapped with a point cloud of the bridge. In this case, the UAV touches the bridge five times and stays in contact for five seconds each time. The measurements of the contact point can be used to estimate the deformation of the bridge and complete the inspection task. This result confirms that inspection is possible with the prototype presented in this work.

### 5.2. Grazalema Bridge Experiments

**Figure 18:**
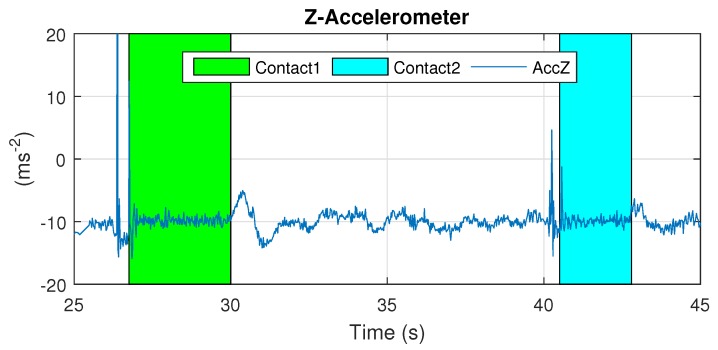
Z-accelerometer—Grazalema bridge experiments.

**Figure 19:**
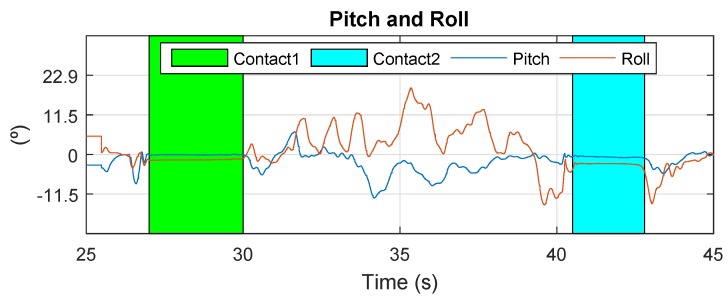
Pitch and roll—Grazalema bridge experiments.

**Figure 20:**
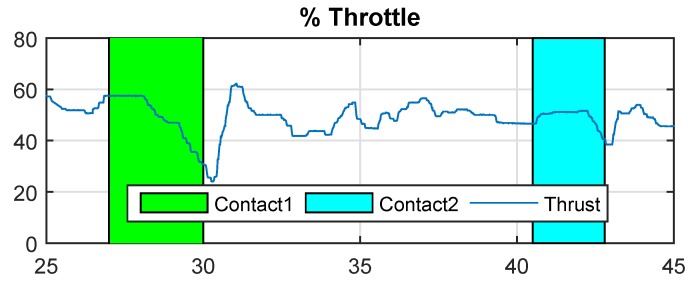
% Throttle—Grazalema bridge experiments.

**Figure 21:**
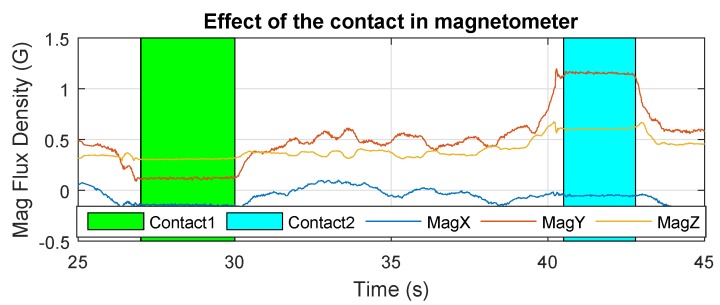
Magnetometer—Grazalema bridge experiments.

**Figure 22:**
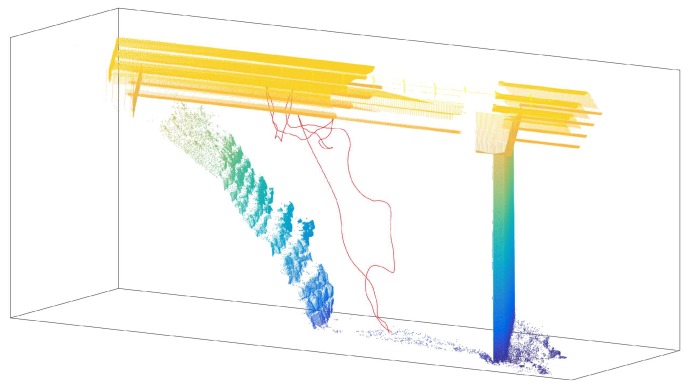
Prism trajectory measured with the laser tracker—Grazalema bridge experiments.

The results presented in this section are the telemetry data collected during the bridge-inspection task at the Grazalema bridge (Figure 10). These results are presented in Figure 18, Figure 19, Figure 20, Figure 21 and Figure 22.

The two contact conditions were different in this case. The first contact (green background) was carried out between two beams, and the second contact occurred at the bottom part of the beam (cyan background). This experiment provides evidence related to two different problems. In the first contact, the aerodynamic problem is more constrained because the UAV needs to fly in a more confined space (second experiment of Reference [32]). However, in the second contact, the available area was narrower, making it difficult to perform the contact with the whole fairing (third sequence of Reference [32]); the cylindrical design of the fairings solves this issue.

The results presented in Figure 18 and Figure 19 show that the effects in these measurements are similar to those shown in previous results presented in Figure 12 and Figure 13. The z-accelerometer is triggered at the start of the contact condition, and the attitude angles remain almost constant during it.

Figure 20 shows that the effect is different in the two contact conditions. It can be clearly seen that the ceiling effect is more significant in the first contact (green background). This is a consequence of carrying out the contact in a span between two beams where airflow is more constrained by the lateral beams. Therefore, the suction force generated by the ceiling effect is stronger and the thrust of the rotors has to be lowered significantly more than in the second case for the UAV to un-stick it from the bridge, as can be seen in the figure.

The measurements of the magnetometer in Figure 21 show that the magnetic-field perturbation effect close to the structure again appears in a different bridge, and it reinforces the need for the changes in the autopilot estimator to occur to avoid dangerous crashes.

As the last result, Figure 22 presents the prism trajectory measured with the total station during a real experiment. The UAV contacts the bridge at four points in this experiment, two points in the span and two in the beam. In the case of an inspection, the number and positions of the points can be determined by the bridge-maintenance engineer.

## 6. Conclusions

This paper proposed an autonomous methodology to carry out bridge-beam inspection by contact using UAVs. The robotic inspection system presented in this paper takes advantage of the aerodynamic ceiling effect that was previously studied by the authors. Moreover, possible drawbacks were analyzed. The design of an aerial platform that meets the inspection requirements and exploits the benefits of this ceiling effect was designed, built, and tested in real bridge-beam inspections.

The presented design allows the aerial platform to safely stick to the bridge. The stability of the platform during contact is improved by customizing the fairings. Furthermore, the maximum flight time is increased using the ceiling effect in favor of the aerial platform. Additionally, the inspection method and the platform design ensure that the movements of the platform are conservative in order to increase the precision of the inspection measurements. Moreover, the presented system focuses on monitoring the state of bridges over time because, thanks to the robotic total station used, it is possible to directly compare the measures of two different inspections due to them both being in the same reference frame.

Future work related to this research will focus on the implementation of a dedicated control system during the contact phase, increasing the system technology readiness level (TRL) and improving the performance of the aircraft in order to autonomously carry out bridge-beam inspections. Moreover, the authors aim to validate the inspection system presented in this paper and to assess the limits of operation by comparing the inspection results with the results obtained by a classical experimental campaign.
